# Long-term prognosis of patients with cardiac syndrome X: a review

**DOI:** 10.1007/s12471-012-0256-z

**Published:** 2012-02-23

**Authors:** I. A. C. Vermeltfoort, G. J. J. Teule, A. B. van Dijk, H. J. Muntinga, P. G. H. M. Raijmakers

**Affiliations:** 1 Department of Nuclear Medicine, Verbeeten Institute, PO Box 90120, 5000 LA Tilburg, the Netherlands; 2 Department of Cardiology, Tweesteden Hospital, Dr Deelenlaan 5, 5042 AD Tilburg, the Netherlands; 3 Department of Nuclear Medicine & PET Research, VU University Medical Centre, PO Box 7057, 1007 MB Amsterdam, the Netherlands

**Keywords:** Cardiac syndrome X, Microvascular angina, Angina pectoris AND normal coronary arteries, Prognosis

## Abstract

**Aims:**

Follow-up studies of patients with cardiac syndrome X (CSX) generally report good prognosis. However, some recent studies report an adverse outcome for women.

**Methods and results:**

Structured literature search and meta-analysis for studies regarding prognosis of cardiac syndrome X patients. We identified 85 studies, ultimately selecting 16 for inclusion. Meta-analysis yielded a pooled major cardiac event percentage of 1.5% per 5 years and a pooled vascular event percentage of 4.8% per 5 years (*n* = 16 studies, *n* = 1694 patients). Fourteen studies reported upon the recurrence rate of angina pectoris: the pooled percentage of angina recurrence was 55% (*n* = 1336 patients).

**Conclusion:**

The present review of recent archival literature demonstrates an overall major cardiac event rate of 1.5% per 5 years. Although this is an excellent prognosis for CSX patients, the quality of life is impaired because of the high recurrence rate of angina pectoris (55%).

## Introduction

Cardiac syndrome X (CSX) is the syndrome of angina pectoris during a positive stress test despite a normal coronary arteriogram. CSX is an important clinical entity [1–19]. About 3–11% of patients undergoing coronary angiography because of typical chest pain have normal coronary arteries and qualify for the definition of CSX [19].

Follow-up studies of patients with CSX generally report good prognosis [7, 9]. However, a recent study reported an adverse cardiovascular outcome for women with chest pain and normal coronary arteries [6].

Therefore, the present paper reviews the recent literature on CSX to discuss in detail the long-term prognosis.

## Methods of identifying and selecting the literature

A review and meta-analysis of the literature was conducted with a comprehensive search of the PubMed database to identify clinical studies on CSX that considered prognosis. The prognosis of CSX patients was derived from the cardiac event rate per year for each study and the recurrence rate of angina (when included in the studies).

Studies were eligible for review if they met the following standardised inclusion criteria [20–22]: Full-length articles; studies including CSX or patients with angina and normal coronary arteries; minimum number of 10 patients; studies presenting follow-up data of more than 2 years; and publication dates between April 1985 and February 2010.

The criteria for excluding studies were: metabolic syndrome X; studies including patients with near-normal coronary angiography (CAG) results; and studies of patients with myocardial infarction and/or cardiomyopathy were excluded.

In detail, the PubMed database was used to identify papers in which definitions and prognosis for CSX are described via the following terms:

‘((Prognosis or follow up) AND ((((cardiac syndrome x OR (angina pectoris AND normal coronary arteries) OR microvascular angina)) AND (((“microvascular angina”[TIAB] NOT Medline[SB]) OR “microvascular angina”[MeSH Terms] OR cardiac syndrome x[Text Word]))) AND ((“syndrome x”[All] OR (((angina pectoris) OR (chest pain)) AND normal AND (coronary[tw] OR angiogra*[tw])) OR (microvascular angina)))) AND ((Humans[Mesh]) AND (English[lang]) AND (adult[MeSH])))’; with limits set to English language and humans.

The search strategy identified 85 studies from PubMed. These became the source population for this review. The titles were screened for eligibility by one of the reviewers (I.V.), followed by two reviewers (I.V. and P.R.) independently assessing the abstracts by consensus. In this initial screening a total of 23 articles were excluded because of:

- Metabolic syndrome X, myocardial infarction and tako-tsubo cardiomyopathy (14 references).
- Non-performance of coronary angiography (2 references).
- Studies with coronary spasm (3), case report (1), antiphospholipid syndrome (1) and non-obstructive coronary artery disease (2) in the title.

The titles of the remaining papers contained cardiac syndrome X (44 papers), normal coronary arteries (14 papers) and microvascular angina (4 papers).

The full texts of all 62 papers were retrieved for further selection, notably as to whether they considered prognosis. Only 17 included prognosis, but checking the references from all 17 resulted in another 4 papers being selected [1, 6, 12, 14]. Of these remaining 21 papers, 9 described CSX, 11 described AP and normal coronary arteries (NCA), and 1 described microvascular angina.

Finally, the 21 papers were independently surveyed by two authors (IV and PR) with respect to the definition of CSX and prognosis. This resulted in 5 papers being excluded as follows: an NCA study [23] also included minimal lesions of the coronary arteries with a reduction in diameter of less than 50%; one turned out to be only a letter [24]; 2 intervention studies did not report cardiac events [25, 26]; and another appeared to be an intervention study of only 7 patients [27], whereas the agreed minimum number of patients for our review was 10.

This survey left 16 papers for the present review. These papers covered a total of 1694 patients. For each paper we extracted the number of patients; cardiovascular event rates; percentage of female/male patients (for studies including both female and male patients); and the percentage of patients with recurrent chest pain. In more detail:

1. Major adverse cardiac event rates included cardiac death, myocardial infarction and revascularisation as defined by the most recent ACC/AHA guidelines 2010 and other studies [28–30].
2. The vascular events included myocardial infarction, cardiac death, development of CAD, heart failure, and the occurrence of cerebrovascular events.
3. Since some of the studies had different follow-up periods, we normalised the event rates to those per 5 years.
4. Before calculation of the pooled estimates of major cardiac event rates, cardiovascular event rates, chest pain recurrence rates and gender distribution, the heterogeneity of these values was tested using calculation of I^2^ and the Chi-square test. The effects of follow-up period, gender distribution and mean age of the populations upon the cardiovascular event rates were studied using regression analysis: P values < 0.05 were considered to be significant.

## Results

Table 1 lists the 16 studies included in this review. Note that (a) 2 studies did not report the frequency of chest pain [3, 6], (b) the follow-up period varied widely, from 2.4 years to 14 years, and (c) 3 studies did not have male and female patients [1, 3, 6].

**Table 1:** Cardiac syndrome X

First author	Year	No	Average age (yr)	% Men	Average FU (yr)	Coronary event			% Chest pain recurrence	% Repeat CAG	
						Death	MI	CAD			
Bugiardini [1]	2004	42	51.6 ± 8.8	0	10.3	1	0	13	31	–	
Chauhan [2]	1993	41	46 ± 4	39	2.9	0	0	0	64	–	
Delcour [3]	2009	48	60.9 ± 12	100	7.4	0	1	5	–	–	6 CVA
Foussas [4]	1998	160	50	69	2.5	1	2	0	59	–	
Fragasso [5]	2009	34	56	20	14 ± 2	0	2	2	65	–	1 LV dysfunction
Gulati [6]	2009	318	53.6 ± 10.4	0	5.2	5	3	0	–	–	8 CVA; 10 LV dysfunction
Kaski [7]	1995	99	48.5 ± 8	21	7 ± 4	0	0	0	89	–	1 LV dysfunction
Lamendola [8]	2008	155	58.9 ± 10	26	11.4 ± 6.5	0	0	3	48	21	
Leu [9]	2005	92	63.9 ± 10.5	78	2.6 ± 1.2	0	0	0	13	9	1 LV dysfunction; 3 CVA
Lichtlen [10]	1995	176	48.3	65	12.4	2	4	8	81	12	
Radice[11]	1995	30	51 ± 6	27	12.3 ± 3.5	0	0	0	33	27	
Scholz [12]	2002	173	54 ± 7.8	59	12.0 ± 2.9	10	0	6	34	–	
Shintani [13]	2003	43	55 ± 8	7	6.4 ± 3.8	0	0	0	44	–	
Sullivan [14]	1994	138	50.0	55	2.4	0	1	0	70	–	
Sun [15]	2001	59	61 ± 5	88	7.1 ± 1.4	0	1	0	76	–	10 LV dysfunction
Suzuki [16]	2002	86	59 ± 9	41	7.2 ± 3.4	0	0	0	35	2	

– = non-available

*CAD* coronary artery disease, *CAG* coronary angiography; *CVA* cerebrovascular accident; *FU* follow-up; *LV* left ventricular; *MI* myocardial infarction

### CSX prognosis

The three most important results relating to prognosis are:

1. The major cardiac event rate varied from 0% to 3.8% per 5 years, with a pooled value of 1.5% per 5 years (95% CI: 1–2.2%, *n* = 1694 patients) (Fig. 1). There was no significant heterogeneity between included studies regarding the major cardiac event rate (*p* = 0.074). The estimated annual major cardiac event rate was 0.3%. This includes myocardial infarction, revascularisation and cardiovascular death (Table 1).

**Fig. 1:**
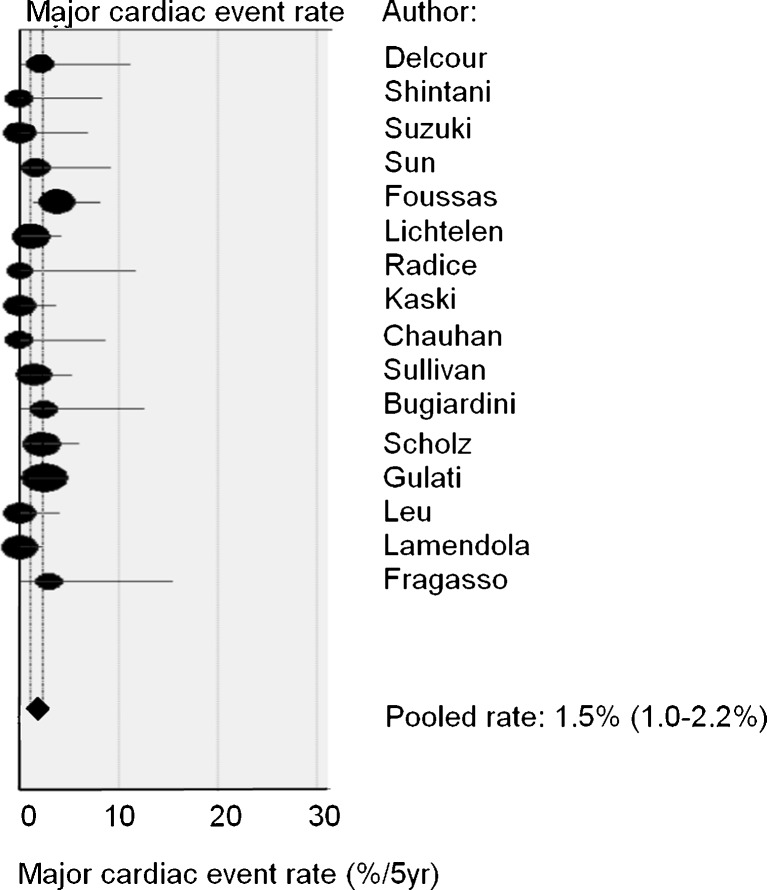
Major cardiac event rate
2. The general cardiovascular event rate varied from 0% to 16.7% per 5 years, with a pooled value of 4.8% for 5 years (95% CI: 3.8–5.9%). However, there was significant heterogeneity (*p* < 0.001) (Fig. 2).

**Fig. 2:**
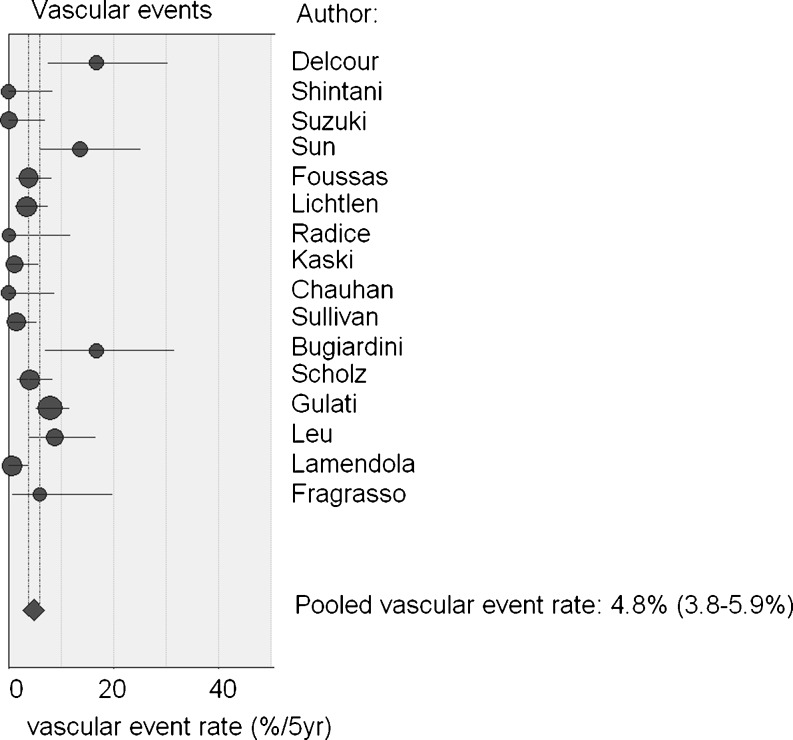
General cardiovascular event rate
3. There were 14 studies with 1336 patients for analysis of recurrent chest pain. The recurrence rate varied between 13.2% and 89.9% for the different study populations, with a pooled value of 55% (95% CI: 53–58%, *n* = 1336 patients). There was significant heterogeneity (*p* < 0.001) (Fig. 3).

**Fig. 3:**
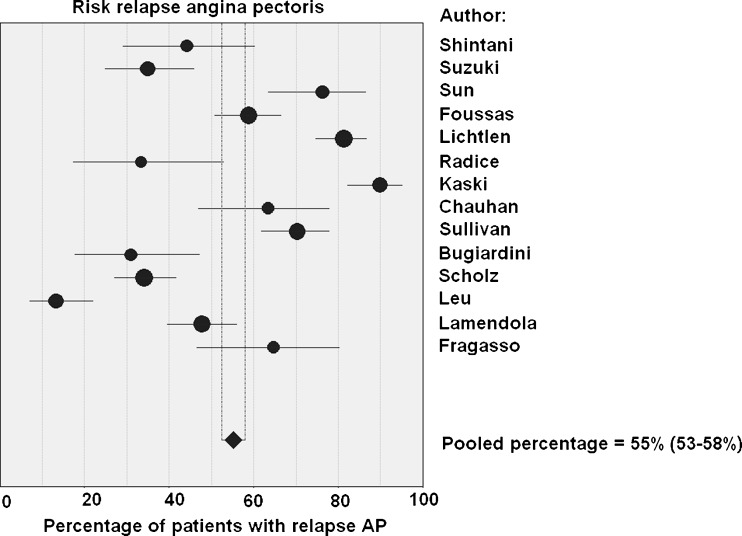
Chest pain recurrence

The remaining results are:

(4) The major cardiac event rate was not related to the length of follow-up (R^2^ = 0.0043), mean age (R^2^ = 0.000006) and percentage of female patients (R^2^ = 0.0268).
(5) The total vascular cardiac event rate per study was not related to the length of follow-up (R^2 =^0.0011), mean age (R^2^ = 0.238) and percentage of female patients (R^2^ = 0.063).

## Discussion

### Overall cardiac event rates

This systematic review shows that the CSX prognosis in terms of the overall cardiac event rate is excellent, with a risk of myocardial infarction or cardiovascular death of 1.5% per 5 years. The Framingham Heart Study reported annual hard (death, myocardial infarction) coronary event rates of 1.2% (men < 65 years); 2.7% (men > 65 years); 0.5% (women < 65 years); and 1.6% (women > 65 years). These rates are all higher than the major adverse cardiac event rate for CSX patients determined from our systematic review.

The lower cardiac event rates for CSX patients suggest that they may benefit from protective factors against coronary macrovascular disease. These factors could be (a) cardioprotective medication, (b) a healthier lifestyle to avoid angina and myocardial infarction, e.g. becoming non-smokers, and (c) another biological mechanism. A possible example of the last mentioned is the observation that platelet reactivity decreases after stress in patients with CSX, in contrast to patients with coronary artery disease (CAD) [31, 32]. On the other hand, the better prognosis of CSX patients compared with the general population may result from a selection process. CSX patients all have, *by definition*, normal coronary arteries, which is not necessarily the case in the general population. The difference in prevalence of coronary atherosclerosis between CSX patients and the general population may explain the difference in cardiac event rates.

The notion of the benign nature of CSX has been challenged by reports of a high risk of future cardiac events in patients with angina and normal coronary arteries [1, 33, 34]. A recent study, named the Women’s Ischaemia Syndrome Evaluation (WISE) study, involved 540 symptomatic women referred for coronary angiography and followed up for a mean of 5.2 years. The control group was asymptomatic, and consisted of community-based age- and race-matched women with no history of heart disease, and who were followed up for 10 years.

The WISE study showed a cardiovascular event rate of 7.9% per 5 years for women with angina and normal coronary arteries (CSX), compared with 2.4% per 5 years for the asymptomatic control group [6]. The WISE study CSX event rate lies outside the 95% confidence interval of 3.8%–5.9% per 5 years that we found from our reviewed studies.

However, the rate of cardiovascular death was not significantly different between the CSX women and the control (asymptomatic) women in the WISE study. The increased cardiovascular event rate in symptomatic women with normal coronary arteries was largely accounted for by an increased incidence of hospitalisation for heart failure and stroke. It remains unexplained why the incidence of heart failure and stroke are increased in this specific population, although microvascular dysfunction might precede macrovascular atherosclerosis.

### CSX and non-obstructive CAD cardiac event rates

It is important to distinguish between CSX, with normal coronary arteries, and non-obstructive CAD [19]. The cardiac event rate for CSX patients is significantly better than that for patients with angina and non-obstructive CAD. This is illustrated by Sicari et al. [35], who reported that a subgroup of patients with angina and non-obstructive CAD (incorrectly identified as CSX), and with positive dipyridamole echocardiography tests, had a survival rate of only 76% after 7.1 years follow-up.

Five-year cardiac event rates for cardiovascular events were significantly different for three subgroups in the WISE study: 16% per 5 years for women with angina and non-obstructive CAD (stenosis <50%); 7.9% per 5 years for women with angina and normal coronary arteries (CSX); and 2.4% per 5 years for the asymptomatic control group (*P* ≤ 0.002) [6]. Hence, in this large study a higher incidence of events in symptomatic women with non-obstructed CAD was found compared with patients with symptoms and normal coronary arteries.

### CSX and coronary microvascular dysfunction

Lanza and Crea recently proposed to rename CSX as stable primary coronary microvascular dysfunction (CMVD) [36]. This proposal was made on the premise that abnormalities in the coronary microcirculation are the probable cause of CSX ischaemia and angina. However, Herzog obtained contemporary positron emission tomography (PET) data that suggest an increased cardiac event rate for patients with microvascular dysfunction, irrespective of abnormalities in the epicardial coronary arteries [37]. (Note: this study was not included in our literature review because CAGs were not performed.)

In the study by Herzog the patients with a reduced flow reserve, defined as a CFR < 2.0, had a higher annual cardiac event rate and a higher risk of cardiac death, and this included patients with a normal perfusion PET [37]. Specifically, a subgroup of patients with normal perfusion but impaired CFR had a significantly higher major annual cardiac event rate (6.25%) compared with patients with a normal CFR (1.4%). Also, the annual cardiac death rate was higher: 3.1% for patients with normal perfusion but impaired CFR, compared with 0.5% for patients with normal CFR. Thus it is possible for patients with a normal perfusion, who are therefore unlikely to have epicardial coronary obstructive disease, to already have a reduced CFR, and that this is associated with a higher cardiac event rate or higher risk of cardiac death. The reduced CFR was most likely caused by microvascular or endothelial dysfunction [37]. Therefore one should be cautious about equating CSX (which has a generally good prognosis) with coronary microvascular dysfunction.

Limitations to the significance of the study by Herzog et al. are (a) the relatively small number of patients in the subgroup with normal perfusion and impaired CFR (*n* = 32), and (b) that after 10 years follow-up the impaired CFR could not predict any cardiac event in this subgroup [37]. Hence larger prospective studies are needed. Be that as it may, we may conclude that a distinguishing diagnosis between CSX and CMVD requires evaluation of the distal compartment (intramural arterioles) as well as the proximal compartment (the large epicardial coronary arteries).

### Recurring chest pain

As stated earlier, recurrence of chest pain occurred in an average of 55% of the CSX cases included in our systematic review. In the study by Lantinga et al., 85% had at least weekly episodes of chest pain up to 1 year after the angiograms, with the pain unchanged or even worse; and 33% underwent at least one more coronary angiography [38].

The most important therapy consists of reassurance, risk factor modification, and symptom relief (ACC/AHA guidelines 2002, www.acc.org/qualityandscience/clinical/statements.htm). There are conflicting data about the exact cause of chest pain in patients with CSX, There is evidence for ischaemia [17, 39], and alternatively, psychological factors such as an increased anxiety may play a role in CSX [18]. The number of pain episodes can be reduced by β-blockers, calcium antagonists, nitrates and imipramine, which is particularly successful [40]. Similar success has been claimed for oestrogen replacement therapy [41, 42]. In a patient with coronary spasms we found an increase of the myocardial blood flow and decrease of symptoms using bosentan, an endothelin receptor antagonist [43]. However, the therapeutic measures remain largely empirical, and the symptoms persist in many patients.

## Limitations

Systematic reviews are hampered by publication bias, i.e., the preferential publication of studies with significant positive results rather than those with negative results. However, in studies regarding prognosis and survival this phenomenon is probably less frequent compared with studies evaluating therapeutic strategies.

Another potential problem is that clinical prognostic studies are almost inevitably different in their length of follow-up, which can affect the study results because longer follow-up periods might yield more exact prognostic figures. However, in our review we found no relation between the length of follow-up and event rates.

There are possible limitations owing to varying inclusion criteria for the different studies. These variations can cause heterogeneous meta-analysis results. For example, we found in earlier work that there were more than 50 different criteria (nine different inclusion criteria and 43 different exclusion criteria) for CSX, resulting in a varying reported incidence of CSX [19]. Also, the heterogeneity of the different study populations may have contributed to the heterogeneity that we found with respect to the prognoses from the studies.

On the other hand, the advantages of reviews and meta-analyses are an increase in statistical power, the ability to assess sources of heterogeneity, and the provision of overall estimates of prognostic variables.

## Conclusions

The present review found an overall major adverse cardiac event rate of 1.5% per 5 years. This represents a better prognosis compared with the general population. However, angina pectoris in CSX is recurrent and persistent in 55% of the patients, and significantly impairs the quality of life.

Whether CSX patients benefit from protective factors against acute coronary events is a challenging issue that should be addressed in future studies.
